# Is recovery driven by central or peripheral factors? A role for the brain in recovery following intermittent-sprint exercise

**DOI:** 10.3389/fphys.2014.00024

**Published:** 2014-02-03

**Authors:** Geoffrey M. Minett, Rob Duffield

**Affiliations:** ^1^School of Exercise and Nutrition Sciences, Queensland University of TechnologyKelvin Grove, Brisbane, QLD, Australia; ^2^Institute of Health and Biomedical Innovation, Queensland University of TechnologyKelvin Grove, Brisbane, QLD, Australia; ^3^Sport and Exercise Discipline Group, UTS: Health, University of Technology SydneyLindfield, Sydney, NSW, Australia

**Keywords:** team-sports, football, soccer, fatigue, central nervous system, contractile function, muscle damage

## Abstract

Prolonged intermittent-sprint exercise (i.e., team sports) induce disturbances in skeletal muscle structure and function that are associated with reduced contractile function, a cascade of inflammatory responses, perceptual soreness, and a delayed return to optimal physical performance. In this context, recovery from exercise-induced fatigue is traditionally treated from a peripheral viewpoint, with the regeneration of muscle physiology and other peripheral factors the target of recovery strategies. The direction of this research narrative on post-exercise recovery differs to the increasing emphasis on the complex interaction between *both* central and peripheral factors regulating exercise intensity *during* exercise performance. Given the role of the central nervous system (CNS) in motor-unit recruitment during exercise, it too may have an integral role in post-exercise recovery. Indeed, this hypothesis is indirectly supported by an apparent disconnect in time-course changes in physiological and biochemical markers resultant from exercise and the ensuing recovery of exercise performance. Equally, improvements in perceptual recovery, even withstanding the physiological state of recovery, may interact with both feed-forward/feed-back mechanisms to influence subsequent efforts. Considering the research interest afforded to recovery methodologies designed to hasten the return of homeostasis within the muscle, the limited focus on contributors to post-exercise recovery from CNS origins is somewhat surprising. Based on this context, the current review aims to outline the potential contributions of the brain to performance recovery after strenuous exercise.

## Introduction

Team sport athletes experience varying levels of transient (acute) and residual (on-going) fatigue during periods of intense training and competition (Borresen and Lambert, 2009). Characterized by repeated bouts of intermittent-sprint exercise, field-based team sports demonstrate high physiological (Spencer et al., 2005a), neuromuscular (Rampinini et al., 2011; Duffield et al., 2012) and perceptual demands (Impellizzeri et al., 2004). These relative stresses are compounded when successive bouts compromise the innate rate of recovery, and subsequently may limit ensuing performance (Bishop et al., 2008). Artificially speeding the natural time course of regenerative processes via recovery strategies is important in preparing for ensuing training or competition bouts (Barnett, 2006). However, an understanding of the mechanisms driving recovery is critical to determine the likely beneficial effects of these interventions; especially given the theoretical framework of team sport performance recovery research remains limited in scope and with specific focus on the periphery (Nédélec et al., 2013).

Various recovery modalities have been advocated to treat exercise-induced muscle damage (EIMD) and associated delayed-onset muscle damage (DOMS), with their efficacy the focus of considerable critical (Barnett, 2006; Bishop et al., 2008; MacRae et al., 2011; Nédélec et al., 2013) and systematic review (Bleakley et al., 2012; Leeder et al., 2012; Bieuzen et al., 2013; Poppendieck et al., 2013). While such recovery strategies might be broadly differentiated as being either physiological (e.g., cryotherapy, hydrotherapy, massage, compression, sleep), pharmacological (e.g., non-steroidal anti-inflammatory medications) or nutritional (e.g., dietary supplements), all mean to limit continued post-exercise disturbances and inflammatory events within the exercised muscle cells. This peripheral focus emphasizes the importance of an accelerated return of structural integrity and functional capacity from below the neuromuscular junction. However, increasing evidence demonstrates disassociated rates of physical and physiological recovery following intermittent-sprint exercise, particularly the return of neuromuscular force despite continued rise in blood-based EIMD markers (Pointon et al., 2012; Minett et al., 2013). Accordingly, greater consideration for the role of the central nervous system (CNS) in the recovery of exercise performance is warranted.

Theoretically, the rate of post-exercise recovery is related to the extent of the load imposed on the various physiological and neuromuscular systems by the exercise bout (Nédélec et al., 2012). As such, it is assumed that the physiological bases of recovery are dependent on restoration or reversal of these stresses at the periphery. Contrastingly, the regulation of performance *during* exercise has been increasingly interpreted as an integrative, multifaceted phenomenon (Knicker et al., 2011; Noakes, 2012) with the brain highlighted as central to this process (Gandevia, 2001). While some debate exists as to whether the regulation of exercise performance are conscious (Marcora, 2008) or anticipatory events (Marino, 2004)—derived from afferent or efferent origins (St Clair Gibson and Noakes, 2004); all highlight changing CNS drive and motor unit recruitment to be associated with fatigue and the reduction of intermittent-sprint exercise performance (Billaut, 2011; Girard et al., 2011). Conceptually, if the brain is held as central to the process of performance declines (i.e., fatigue), it stands to reason that it would also have some role in post-exercise recovery (De Pauw et al., 2013). Accordingly, this review aims to discuss the recovery of the CNS in conjunction with the periphery after intermittent-sprint exercise, as encountered in team sport exercise.

## Fatigue and the need for recovery after intermittent-sprint exercise

In highlighting the above point, it is important to first identify the need for recovery before discussing potential drivers from central (neural/brain) and peripheral (muscular) viewpoints. Classically defined as an exercise-induced reduction in force generating capacity of the muscle, fatigue may be attributed to peripheral contractile failure, sub-optimal motor cortical output (supraspinal fatigue) and/or altered afferent inputs (spinal fatigue) innervating the active musculature (Gandevia, 2001). Separately, laboratory simulations of intermittent-sprint exercise have been utilized to examine fatiguing mechanisms related to team sports (Pointon et al., 2012; Minett et al., 2013) and report an operational definition of fatigue to be associated with a decline in peak sprint speed or power output between multiple efforts (Bishop, 2012). Notably, however, observations of fatigue, or at least reductions in exercise intensity, in field-based environments are more abstract. Time-motion analyses highlight the adoption of pacing strategies during team sports and suggest both “transient” reductions in work rate after intense efforts and “cumulative” declines in distances/speeds covered as matches progress (Mohr et al., 2003; Duffield et al., 2009; Coutts et al., 2010; Akenhead et al., 2013).

While peripheral perturbations in skeletal muscle, cardiovascular functioning and metabolic strain are traditionally linked with transient fatigue (Bangsbo et al., 2006), consideration of CNS contributions to altered pacing strategies are increasingly common (Roelands et al., 2013). Indeed, team sport athletes may briefly alter their movement patterns in response to both competition contexts and accumulating physiological demands, so to preserve high-intensity efforts as a match progresses (Bradley and Noakes, 2013). Alternatively, concepts of residual fatigue remain predominately within the domain of peripherally driven mechanisms, such as blood flow, muscle glycogen repletion and clearance of metabolic wastes (Bangsbo et al., 2006). As discussed in a recent review of recovery in soccer, Nédélec et al. (2012) highlighted post-match declines in sprint time, jump height and maximal voluntary contraction (MVC) force by as much as 36% for up to 96 h post-match. Incomplete recovery before ensuing exercise bouts is likely to be detrimental to intermittent-sprint (Spencer et al., 2005b) and sport-specific skill performance (Minett et al., 2012a), though time-course changes in physical recovery and its respective central and peripheral origins remain unclear (Bishop et al., 2008).

The physical and biochemical changes observed during intermittent-sprint exercise have traditionally been interpreted in terms of metabolic capacity (Glaister, 2005). Indeed, lowered phosphocreatine concentrations (Dawson et al., 1997), reduced glycolytic regeneration of ATP (Gaitanos et al., 1993) and increasing H^+^ accumulation (Bishop et al., 2003) have all been associated with declining intermittent-sprint performance. While reductions in muscle excitability after intermittent-sprint exercise have also been observed (Bishop, 2012), metabolic perturbations are rapidly recovered within minutes (Glaister, 2005). Accordingly, where sustained reductions in performance are experienced (>2 h) across consecutive training or competitive sessions, EIMD has been suggested to be the limiting factor of peak intermittent-sprint power output (Twist and Eston, 2005). This hypothesis is logical, yet limited by an apparent disconnect between the expression of muscle damage markers and the recovery of neuromuscular function following intermittent-sprint exercise (Pointon et al., 2012; Minett et al., 2013). Furthermore, given the effect of subsequent DOMS on altering neuromuscular recruitment or activation patterns (Cheung et al., 2003), it would appear that the focus of recovery should not be that of the periphery alone (De Pauw et al., 2013).

The ultimate indicator of post-exercise recovery is the ability of the muscle to produce force i.e., performance outcomes. Optimal sequencing and intensity of muscle activation and recruitment patterns are key to the development of peak power output (Ross et al., 2001), and so are proposed as important contributing regulators of sustained intermittent-sprint performance (Girard et al., 2011). Reductions in EMG signals following simulated soccer performance appear to align with reduced MVC, and suggest an inhibition of neural drive to remain evident 24 h post-exercise (Rampinini et al., 2011). While some caution is advised when directly interpreting surface EMG during dynamic activity (Farina, 2006), declines in voluntary activation (VA) determined using the twitch interpolation method after repeated-sprint activity have been reported (Racinais et al., 2007; Perrey et al., 2010). Notably, field-based findings are mixed, with impaired VA apparent in some (Girard et al., 2008, 2010), but not all intermittent-sprint based sports following competitive matches (Duffield et al., 2012). These discrepancies are likely explained by both the type of sport i.e., task-specific load encountered, as well as methodological differences. Specifically, changes in neuromuscular function following exercise are affected by the measurement duration (Girard et al., 2013) and proximity to the conclusion of the exercise bout (Froyd et al., 2013). Accordingly, it is conceivable that neuromuscular fatigue may be underestimated in field-based settings (Froyd et al., 2013), particularly where immediate access to participants post-match can be difficult.

In summary, performance related declines in intermittent-sprint performance appear to be a multifaceted phenomenon stemming from neuromuscular, contractile and metabolic pathways (Girard et al., 2011; Bishop, 2012). While the inclusion of advanced neurological measures have provided valuable insight into the contributions of central and peripheral fatigue incurred immediately after intermittent-sprint exercise (Girard et al., 2013), understanding of the time-course recovery of these mechanisms remains to be fully elucidated. Further, considering the specificity of physiological responses to a given exercise task, increased understanding of recovery kinetics following intermittent-sprint exercise is required, particularly in field-based environments. The role of the brain, and so the CNS, has been widely acknowledged as integral in regulating self-paced exercise (Tucker, 2009; Roelands et al., 2013), although what role the brain plays in recovery has not been well considered.

## Peripheral recovery after intermittent-sprint exercise

Changes in physical performance following prolonged intermittent-sprint exercise are best represented by reductions in peak speed, total distance covered and high-intensity running efforts (Mohr et al., 2003; Coutts et al., 2010). These changes in field-based performance markers are temporally aligned with reductions in skeletal muscle function, inferred from observations of reduced MVC and CMJ performance (Cormack et al., 2008; McLellan et al., 2011; Duffield et al., 2012). Reductions in skeletal muscle function after intermittent-sprint exercise are often proposed to be caused by a range of peripherally-induced factors, including: intra-muscular glycogen depletion; increased muscle and blood metabolites concentrations; altered Ca^++^ or Na^+^-K^+^ pump function; increased skeletal muscle damage; excessive increases in endogenous muscle and core temperatures; and the reduction in circulatory function via reduced blood volume and hypohydration (Duffield and Coutts, 2011; Bishop, 2012; Nédélec et al., 2012). As outlined by Coutts et al. (in press), either collectively or in isolation, many of these exercise-induced alterations in physiological state reduce post-exercise force production and are proposed to affect the ensuing rate of performance recovery. It is assumed that alleviating these physiological perturbations would facilitate the return of neuromuscular contractile force, and so intermittent-sprint performance.

The glycolytic and oxidative strain incurred during intermittent-sprint exercise is marked, with considerable declines in energy substrate availability acknowledged as contributing to reduced skeletal muscle force production (Girard et al., 2011). Accordingly, limitations in energy supply, be it inorganic phosphate or stored muscle glycogen concentrations, are also thought to hinder recovery after team sport activity (Nédélec et al., 2012). Although phosphocreatine concentration (depleted to 35–55% of resting following a 6 s sprint effort) is associated with power output (Bishop, 2012), the brief and variable recovery periods experienced in team sports do not always allow complete resynthesis to occur. Regardless, given the timeline of phosphocreatine regeneration, it is not considered a factor in recovery for competitive or training bouts separated by hours or days. Conversely, Krustrup et al. (2006) reported declines in intramuscular glycogen of 42 ± 6% in soccer players, with depleted or almost depleted glycogen stores in ~55% of type I fibers and ~25–45% of type II fibers reasoned to explain acute declines in sprint speed post-match. Importantly, muscle glycogen resynthesis after team sport activity is slow and may remain attenuated for 2–3 days (Nédélec et al., 2012). Such findings highlight the importance of nutrition in post-exercise recovery (Burke et al., 2006); yet it is noteworthy that muscle glycogen stores remain impaired 24 h after a soccer match, irrespective of carbohydrate intake and should be recognized as a factor in sustained post-match suppression of force (Bangsbo et al., 2006; Krustrup et al., 2011).

**Figure 1:**
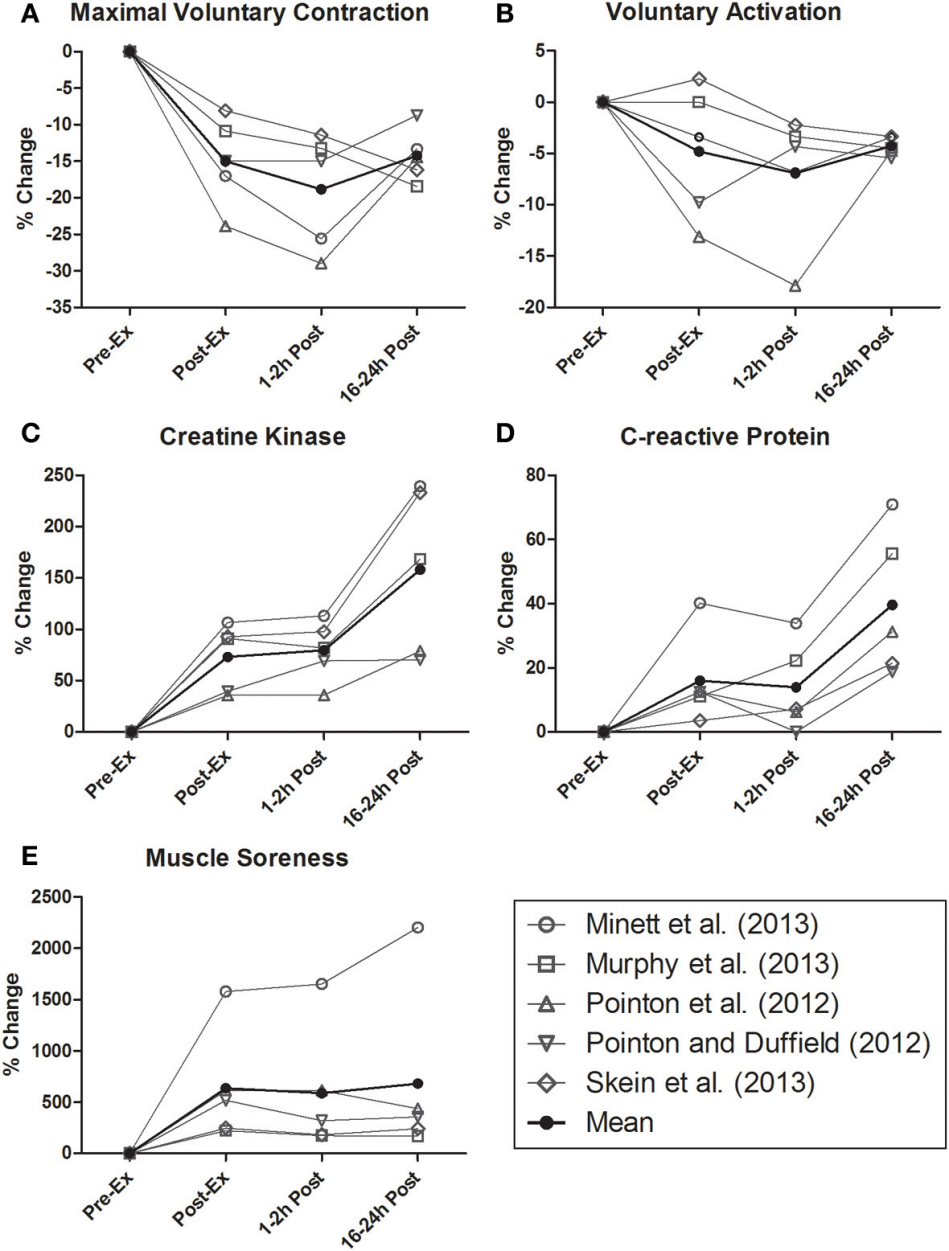
**Time-course change in (A) maximal voluntary contraction of leg extensors, (B) voluntary activation of leg extensors, (C) creatine kinase, (D) C-reactive protein, and (E) perceived muscle soreness following prolonged intermittent-sprint exercise**. These data are redrawn from the studies of Pointon and Duffield (2012), Pointon et al. (2012); Skein et al. (2013), Minett et al. (2013), and Murphy et al. (2013). A collated mean value derived from each of the aforementioned studies has been calculated and is also presented.

In addition to the compromised energy substrate availability/supply experienced following intermittent-sprint activity, repeated high-force eccentric contractions incurred during team sport exercise disturb skeletal muscle function and evoke marked muscle damage responses (McLellan et al., 2011). Mechanical disruptions to the muscle fiber are task dependant, though likely relate to the volume of acceleration, deceleration, directional change and inter-player contact completed (i.e., tackling or collisions) (McLellan et al., 2011; Duffield et al., 2012). Importantly, EIMD manifests in reduced voluntary force production that has been associated with the elevated expression of intracellular proteins (e.g., creatine kinase and C-reactive protein), swelling, restricted range of motion and muscle soreness (Cheung et al., 2003). Whilst it is generally accepted that lowering blood-based muscle damage profiles may hasten athletic recovery, mechanisms explaining the return of skeletal muscle function are somewhat ambiguous (Howatson and Van Someren, 2008). As demonstrated in Figure 1, time-course changes in markers associated with the recovery of voluntary force following intermittent-sprint exercise do not mirror blood-based measures. Interestingly, markers of EIMD are also not closely associated with muscle soreness (Nosaka et al., 2002; Prasartwuth et al., 2005), though perceptual recovery is reportedly related with the recovery of maximal sprint speed (Cook and Beaven, 2013). While this raises questions in terms of the physiological underpinnings of muscle soreness, weaker relationships between EIMD and neuromuscular performance may suggest the potential for other drivers of recovery outside of peripheral (muscle damage or metabolic) factors alone.

Finally, while the relationship between hydration status and intermittent-sprint performance remains contentious (Edwards and Noakes, 2009), fluid deficits of 2–4% are common following team-sport exercise (Duffield and Coutts, 2011). Mild hypohydration reportedly demonstrates limited effects on anaerobic power and vertical jump performance (Hoffman et al., 1995; Cheuvront et al., 2006); however, some caution is required in interpreting these data as these testing protocols reflect only select components of team sport performance. In particular, the detrimental effects of high cardiovascular strain (i.e., hydration-related reduction in plasma volume) in reducing self-paced exercise performance (Périard et al., 2011) may well be applicable to prolonged team sport events. Considering the emphasis that is placed on post-match fluid replenishment (Reilly and Ekblom, 2005), the effect of hydration status on recovery should be negligible wherever an extended period exists between consecutive sessions (Nédélec et al., 2012). Nevertheless, the role of hydration in recovery should not be overlooked as changes in extracellular osmolarity are suggested to influence glucose and leucine kinetics (Keller et al., 2003). Further, the negative psychological associations (conscious or otherwise) derived from a greater perceptual effort incurred in a hypohydrated state may impact mental fatigue (Devlin et al., 2001; Mohr et al., 2010). That said, few studies report the post-match return to euhydration (or from hypohydration) alongside any measure of intermittent-sprint or team-sport related performance.

In summary, the physiological perturbations incurred within peripheral skeletal muscle during prolonged intermittent-sprint exerciseare marked and varied (Bangsbo et al., 2006; Duffield and Coutts, 2011; Nédélec et al., 2012). While many of the peripheral factors classically linked with fatigue during acute performance appear to promptly resume homeostasis, the interrelationships between muscle glycogen depletion, EIMD and hydration may be prolonged and of concern to athletes undertaking back-to-back sessions or competition. Nevertheless, corresponding rates of change in recovery markers (e.g., MVC, CK, CRP, and MS) have not been shown to align, whilst markers of hydration and post-match recovery of performance are interestingly absent from the literature. Accordingly, it seems that despite the common focus of recovery interventions on these markers, no singular factor may represent a direct causative mechanism explaining the rate of return of physical performance. Rather, that the integrative regulation of whole body disturbances based on these peripheral factors, alongside central regulation may be relevant.

## Recovery of CNS function after intermittent-sprint exercise

Although the decline in CNS drive following intermittent-sprint exercise is purported as the major inhibitor of post-match sprint and MVC performance (Rampinini et al., 2011), the relative invasiveness and time constraints surrounding the neurophysiological measures [e.g., computed tomography (CT), magnetic resonance imaging (MRI), transcranial magnetic stimulation (TMS); electroencephalography (EEG) and near-infrared spectroscopy (NIRS)] restrict applied use. Accordingly, pseudo measures of CNS function, such as EMG and estimates of VA via peripheral or cortical stimulation, are increasingly utilized methodologies—particularly within simulated team-sport protocols in laboratory settings (Pointon et al., 2012; Girard et al., 2013; Minett et al., 2013). These measures offer inferred estimates of CNS function, mainly muscular recruitment and motor neuronal signaling to optimally innervate muscle fibers (Gandevia, 2001). Whist the purpose of this discussion is to not review these as measures *per se*, any attempt to understand the role of the CNS in recovery needs to draw on inferences made from such data. Few studies directly evaluate cerebral function following prolonged intermittent-sprint exercise and this presents an area for future investigation (Minett et al., 2013).

Surface EMG is a common methodology used to examine neuromuscular recruitment patterns during exercise and may be interpreted as a surrogate measure of descending motor command (Billaut, 2011). Notably, a direct correlation exists between declining EMG amplitudes and work output during repeated 5 s cycle sprints with minimal recovery (<30 s) (Billaut and Smith, 2010). Similarly as a parallel, alterations in pacing strategies during prolonged endurance exercise seemingly correspond with changes in motor unit recruitment inferred via EMG signaling (St Clair Gibson et al., 2001; Billaut et al., 2013). Considering the neuromuscular demands of team sports (Cormack et al., 2008; McLellan et al., 2011), and evidence of pacing to preserve high-intensity efforts (Coutts et al., 2010; Skein et al., 2011), it might be presumed that recovery of physical performance following intermittent-sprint performance is affected by ensuing muscle recruitment patterns. Analysis of EMG activity [root mean square (RMS)] during knee extensor MVCs following simulated intermittent-sprint team sport protocols, whilst not specific of teams sport demands, have demonstrated post-exercise declines in motor unit activity to be sustained for between 1 and 24 h (Pointon and Duffield, 2012; Pointon et al., 2012; Minett et al., 2013). These findings appear to corroborate the data of Rampinini et al. (2011), suggesting a relationship between impaired MVC and sprint performance with next-day EMG activity (RMS and RMS/PPA ratio) after a 90 min soccer match. Importantly, the lack of change reported in M-wave characteristics after self-paced intermittent-sprint exercise (Rampinini et al., 2011; Minett et al., 2013) is indicative of preserved excitation at the sarcolemma (Allen et al., 2008) and seemingly further implicates a role for central mechanisms in the regulation of post-exercise recovery.

Although there are considerable confounding peripheral factors that may affect the transmission and propagation of the EMG signal (Farina, 2006), peripheral nerve stimulation has been used to assess inferences of central contributions to recovery from muscle fatigue after self-paced intermittent-sprint (Skein et al., 2011; Pointon and Duffield, 2012; Pointon et al., 2012; Minett et al., 2013) and team sport exercise (Rampinini et al., 2011; Duffield et al., 2012; Murphy et al., 2013; Skein et al., 2013). Using this approach, we and others have reported mixed findings, with alterations in VA during repeated (i.e., 8–15) isometric MVC efforts, demonstrating limited (Duffield et al., 2012; Skein et al., 2012b) and varying durations of acute (1–2 h) (Pointon et al., 2012; Minett et al., 2013) and prolonged activation deficit (24 h) (Rampinini et al., 2011; Pointon and Duffield, 2012). These alterations in VA appear task specific to the exercise load and environment encountered; nevertheless, under conditions of exercise-induced fatigue, a sustained inability of the CNS to optimally drive skeletal musculature during ensuing voluntary efforts may have a detrimental effect on the recovery of intermittent-sprint performance (Rampinini et al., 2011; Bishop, 2012).

As observational evidence of this, Figure 1 highlights the time course of post-exercise recovery in MVC, VA, and relevant markers of muscle damage in the 24 h following prolonged intermittent-sprint exercise (Pointon and Duffield, 2012; Pointon et al., 2012; Minett et al., 2013; Murphy et al., 2013; Skein et al., 2013). As noted, the U-shaped time course of post-exercise MVC measures represents the suppression and regeneration of muscle contractile force. Interestingly, this is temporally aligned with the suppression and recovery of VA. Conversely, similar observations are not evident in muscle damage or perceptual soreness markers. That said, the absence of numerous other potential markers of physiological recovery from this figure must be acknowledged. Regardless, the role of the brain in recovery remains unclear, as VA estimated via peripheral nerve stimulation is proposed to reflect spinal but not necessarily supraspinal output (i.e., motor cortex) (Taylor, 2009). Whilst not team sport specific, relevant literature highlights submaximal corticomotor outflow determined via TMS to contribute to reduce MVC 4 h following a simulated marathon. These declines in descending motorneuronal drive were proposed as an explanation for the observed reduction in central twitch torque (Ross et al., 2009). However, future investigations including the collective use of TMS, EEG and NIRS are required to confirm changes in motor neuronal activity as a factor in neuromuscular recovery following self-paced intermittent-sprint exercise (De Pauw et al., 2013; Minett et al., 2013).

Despite the abovementioned suggestion of a role for increased central regulation being responsible for leading the recovery of skeletal contractile functioning (i.e., after exercise), there remains a paucity of direct evidence to substantiate these claims. In particular, the use of EEG measures to highlight altered cerebral functioning as evidence for changes in the time course of post-exercise recovery has received limited attention (De Pauw et al., 2013). Certainly, temporary increases in EEG alpha activity observed during and after exercise have been linked with reduced cortical activation that may be indicative of central fatigue (Kubitz and Mott, 1996; Crabbe and Dishman, 2004). Notably, De Pauw et al. (2013) reported cold-water immersion (CWI) following prolonged cycling in the heat to hasten the recovery of reduced beta activity in brain areas associated with somatosensory activities. While increased regional brain processing within the insular cortex (BA 13) and supramarginal gyrus (BA 40) are interpreted as reflecting higher arousal, CWI demonstrated no performance benefit in subsequent time trial performance 1 h post-exercise (De Pauw et al., 2013). Consequently, differentiating the emotional or somatic response to the cold water sensation (Schneider et al., 2010) during post-exercise interventions from any increased cognitive function related to recuperative mechanisms remains difficult. Nevertheless, given the focus of the De Pauw et al. (2013) study was to examine the brain functioning and recovery in cyclists, rather than performance based markers, the use of multiple neurophysiological measures (e.g., EEG, EMG, NIRS, and TMS) during longer recovery intervals presents as an area for future research.

Whilst ever the role of the brain as a contributor to neuromuscular recovery (as required following competition and training) remains an open question, it stands to reason that any decline in physical capacity during consecutive bouts of team sport activity may still be explained by peripheral models of exercise performance. After all, disturbances in pre-exercise physiology (e.g., core temperature and glycogen stores) are reflected in altered neuromuscular recruitment patterns and decreased performance outcomes (Bangsbo et al., 2006; Skein et al., 2012b). However, therein lays the issue, as central and peripheral perturbations following exercise (including circulatory, metabolic and thermodynamic responses) purported as detrimental to acute performance promptly return to within normal homeostatic ranges, and thus do not always align with delayed returns in physical capacity. Such events may reason EIMD as the regulating means of performance recovery (Howatson and Van Someren, 2008; Twist and Eston, 2009), though it is just as plausible that accompanying increases in CNS sensitive cytokine release (IL-1, IL-6, and TNFα) might augment fatigue sensations (Ament and Verkerke, 2009). Yet to be discussed, though highly plausible are the potential alterations in *perceived* recovery, which in turn may affect neural physiology highlighting post-exercise recovery as reliant on brain-derived emotion (Noakes, 2012). However, no research studies to date successfully isolate perceptual responses to recovery as mitigating of improved performance; although placebo effects reported with many proposed ergognic aids may add weight to the role of perceived recovery in athletic performance. Interestingly, as noted in Figure 1 the suppression and recovery of MVC was not aligned or seemingly related to the rise in perceived muscle soreness. Admittedly the question of perceived soreness and perceived recovery are not the same, and future research should include explicit scales related to recovery i.e., REST-Q (Kellmann and Klaus-Dietrich, 2000).

Although evidence presented highlights the disconnect between inferred CNS function (i.e.,VA) from many peripheral physiological markers (i.e., EIMD markers) during post-exercise recovery, explicit evidence is admittedly lacking. However, within this framework further investigation in extreme environments may assist to clarify this argument, particularly where external stressors exacerbate exercise-induced demands placed upon the CNS. Exercise in extreme environments (i.e., heat or hypoxia) are well known to reduce intermittent-sprint exercise performance (Drust et al., 2005; Billaut et al., 2013). Such environments are purported to compound not only the physiological responses to exercise, but also to invoke earlier and greater central down-regulation of skeletal muscle recruitment (Tucker et al., 2006; Noakes and Marino, 2007). For example, intermittent-sprint exercise in or with greater exogenous and endogenous heat loads result in reductions in distance covered, alongside greater reductions in post-exercise MVC and VA than cooler physiological states (Minett et al., 2011, 2012b; Skein et al., 2012a). Interestingly, amelioration of that excessive endogenous heat load results in maintenance of MVC and VA with better exercise performance (Minett et al., 2011, 2012b; Skein et al., 2012a). Similarly, removal of that endogenous heat load following exercise is also reported to hasten the recovery of VA and skeletal muscle recruitment following intermittent-sprint exercise in the heat (Pointon et al., 2012; Minett et al., 2013).

Two things are evident from the above literature; firstly, that in extreme environments there is a greater role for central regulation in the manifestation of post-exercise fatigue; and secondly that that removal of the associated effects of the extreme environment by external recovery interventions speeds the recovery of neuromuscular function via improved centrally-mediated functioning (Pointon et al., 2012; Minett et al., 2013). Accordingly, extreme environments (e.g., heat) highlight a context whereby recovery might be mediated as much by changes within the CNS as the restoration of peripheral physiological state (De Pauw et al., 2013). Recovery interventions aimed at improving CNS function may also be more effective in these conditions and have greater impact in athlete preparedness for ensuing exercise bouts. Whether such improvement is mediated by reversal of the physiological state to then promote increased CNS recruitment (i.e., feedback system), or whether it is the improvement in the physiological state of the brain that then promotes increased CNS recruitment of the periphery remains unknown. Interventions such as cold-water immersion following exercise in the heat may affect CNS functioning via removal of the heat load alongside preservation of the peripheral physiological state. Conversely, interventions aimed at the provision of fluid and appropriate nutritional aids i.e., branched-chain amino acids thought to promote brain function (Blomstrand, 2006) may indeed directly act upon the brain to improve ensuing skeletal muscle recruitment to improve recovery. Such questions remain to be elucidated, though would assist to better inform recovery practices in the field.

## Conclusion

Prolonged intermittent-sprint exercise incurs a high physiological, metabolic, neuromuscular and perceptual demand. Much attention has been directed toward understanding the time course of recovery as well as proposed interventions aimed to accelerate the speed of performance return. While peripheral perturbations including EIMD, glycogen depletion and hydration status are detrimental to contractile force, different rates of performance recovery and return of physiological markers suggest recovery cannot be explained by peripheral factors alone. Current understanding of the effects of intermittent-sprint exercise and recovery on the brain remains unclear; though given the observed attenuation in VA and altered neuromuscular recruitment patterns, the contribution of the CNS should be considered important to the recovery process. Accordingly, although speculative, this interaction of biochemical, neurological and central perturbations following strenuous intermittent-sprint exercise would support anecdotal understanding of innate recovery as intensity- and duration-specific. Nevertheless, further data is required so to delineate the central and peripheral drivers of recovery following intermittent-sprint exercise and so plan and structure appropriate and evidence-based recovery interventions.

### Conflict of interest statement

The authors declare that the research was conducted in the absence of any commercial or financial relationships that could be construed as a potential conflict of interest.
